# Novel Proteome Targets Marking Insulin Resistance in Metabolic Syndrome

**DOI:** 10.3390/nu16121822

**Published:** 2024-06-10

**Authors:** Moritz V. Warmbrunn, Harsh Bahrar, Nicolien C. de Clercq, Annefleur M. Koopen, Pieter F. de Groot, Joost Rutten, Leo A. B. Joosten, Ruud S. Kootte, Kristien E. C. Bouter, Kasper W. ter Horst, Annick V. Hartstra, Mireille J. Serlie, Maarten R. Soeters, Daniel H. van Raalte, Mark Davids, Evgeni Levin, Hilde Herrema, Niels P. Riksen, Mihai G. Netea, Albert K. Groen, Max Nieuwdorp

**Affiliations:** 1 Department of Internal and Vascular Medicine, Amsterdam University Medical Centers, Meibergdreef 9, 1105 AZ Amsterdam, The Netherlands; m.v.warmbrunn@amsterdamumc.nl (M.V.W.); n.c.declercq@amsterdamumc.nl (N.C.d.C.); p.f.degroot@amsterdamumc.nl (P.F.d.G.); r.s.kootte@umcutrecht.nl (R.S.K.); a.k.groen@amsterdamumc.nl (A.K.G.); 2 Amsterdam UMC, Gastroenterology and Hepatology, Amsterdam Gastroenterology Endocrinology Metabolism, University of Amsterdam, Meibergdreef 9, 1105 AZ Amsterdam, The Netherlands; 3 Amsterdam UMC, Cardiovascular Sciences, Amsterdam Cardiovascular Sciences, University of Amsterdam, Meibergdreef 9, 1105 AZ Amsterdam, The Netherlands; 4 Department of Internal Medicine, Radboud University Medical Center, 6525 EP Nijmegen, The Netherlands; harsh.bahrar@radboudumc.nl (H.B.);; 5 Department of Endocrinology and Metabolism, Amsterdam UMC, University of Amsterdam, Meibergdreef 9, 1105 AZ Amsterdam, The Netherlands; 6 Diabetes Center, Department of Endocrniology and Metabolism, Amsterdam UMC, VU University Medical Centers, De Boelelaan 1117, 1081 HV Amsterdam, The Netherlands; 7 Amsterdam Cardiovascular Sciences, VU University, De Boelelaan 1117, 1081 HV Amsterdam, The Netherlands

**Keywords:** insulin resistance, obesity, proteomics, tumor necrosis factor, Interleukin-1

## Abstract

Context/Objective: In order to better understand which metabolic differences are related to insulin resistance in metabolic syndrome (MetSyn), we used hyperinsulinemic–euglycemic (HE) clamps in individuals with MetSyn and related peripheral insulin resistance to circulating biomarkers. Design/Methods: In this cross-sectional study, HE-clamps were performed in treatment-naive men (n = 97) with MetSyn. Subjects were defined as insulin-resistant based on the rate of disappearance (Rd). Machine learning models and conventional statistics were used to identify biomarkers of insulin resistance. Findings were replicated in a cohort with n = 282 obese men and women with (n = 156) and without (n = 126) MetSyn. In addition to this, the relation between biomarkers and adipose tissue was assessed by nuclear magnetic resonance imaging. Results: Peripheral insulin resistance is marked by changes in proteins related to inflammatory processes such as IL-1 and TNF-receptor and superfamily members. These proteins can distinguish between insulin-resistant and insulin-sensitive individuals (AUC = 0.72 ± 0.10) with MetSyn. These proteins were also associated with IFG, liver fat (rho 0.36, *p* = 1.79 × 10^−9^) and visceral adipose tissue (rho = 0.35, *p* = 6.80 × 10^−9^). Interestingly, these proteins had the strongest association in the MetSyn subgroup compared to individuals without MetSyn. Conclusions: MetSyn associated with insulin resistance is characterized by protein changes related to body fat content, insulin signaling and pro-inflammatory processes. These findings provide novel targets for intervention studies and should be the focus of future in vitro and in vivo studies.

## 1. Introduction

Metabolic Syndrome (MetSyn) is a combination of risk factors for type 2 diabetes (T2D) [1] and cardiovascular diseases (CVD) [2]. These risk factors include central obesity, dyslipidemia, hypertension, and increased fasting glucose. MetSyn prevalence is rapidly increasing, as it is estimated that one third of adults in the United States meet MetSyn criteria [3]. This increase in the number of individuals with MetSyn will also result in an escalation of T2D incidence, as the relative risk of developing T2D for individuals with MetSyn ranges between 3.53 and 5.17 based on a meta-analysis of 16 multiethnic cohort studies depending on ethnicity and study cohort [1].

Insulin resistance can be defined as an inadequate physiologic response to endogenous or exogenous insulin in insulin-target tissues such as the liver, adipose tissue and skeletal muscles which results in the increased secretion of insulin by beta cells [4]. There are several factors that play a role in the development of increased fasting plasma glucose levels and insulin resistance in MetSyn. Excess visceral fat due to high caloric intake and a sedentary lifestyle are associated with small particle LDL and HDL, increased LDL and VLDL concentrations and insulin resistance [5]. Increased insulin resistance can result in relative hypoinsulinemia when beta cells are unable to compensate for insulin resistance, leading to hyperglycemia. This in turn stimulates the lipolysis of triglycerides, resulting in increased circulating free fatty acids [6]. An increased flux of free fatty acids to peripheral tissue increases inflammation and oxidative stress [7]. Inflammatory mediators such as C-reactive protein (CRP), monocyte chemotactic protein-1 (MCP-1) and interleukins (IL) are upregulated in obesity and have been suggested to contribute to the development of insulin resistance and the associated increased risk for atherosclerotic cardiovascular disease [8]. Furthermore, free radical overload causes systemic oxidative stress and has been strongly related to insulin resistance, independent of BMI [9]. In fact, chronic hyperglycemia, dyslipidemia and oxidative stress contribute to low-grade inflammation and insulin resistance [10].

Due to the metabolic perturbations that are associated with excessive visceral fat accumulation, obesity is considered a “chronic relapsing progressive disease process” [11]. However, up to 30% of individuals with obesity do not seem to develop metabolic perturbations, as seen in MetSyn and the concept of metabolically healthy obesity has previously been postulated [12]. As of now, it is still unclear if metabolically healthy obesity is a transient phase preceding MetSyn, or a stable phenotype in some individuals with obesity. In order to develop efficient treatment strategies against MetSyn, and the progression of MetSyn to T2D, it is necessary to understand which metabolic changes mark the progression from overweight and obesity towards MetSyn.

To better understand which metabolic changes drive insulin resistance in MetSyn, we tested euglycemic hyperinsulinemic clamps in treatment-naive men with MetSyn and evaluated metabolic differences between insulin-resistant and insulin-sensitive individuals with MetSyn. Additionally, we replicated our findings in a cohort of obese subjects with and without MetSyn and hypothesize that impaired fasting glucose (IFG) is associated with a specific metabolic pathway and adipose tissue volume.

## 2. Materials and Methods

### 2.1. Study Design

#### 2.1.1. Discovery Cohort

The discovery metabolic profile study (MPS) cohort was pooled from several fecal microbiota transplantation studies with Caucasian males with MetSyn defined as meeting three or more criteria of the National Cholesterol Education Program (NCEP) Adult Treatment Panel (ATP III), such as fasting glucose ≥ 5.6 mmol/L, waist circumference ≥ 102 cm, blood pressure > 130/85 mmHg, triglyceride levels ≥ 1.7 mmol/L and high-density-lipoprotein (HDL) cholesterol < 1.0 mmol/L [13]. All participants also had a BMI > 28 kg/m^2^. Exclusion criteria included any medication use, comorbidities and history of cholecystectomy, and a comprehensive overview of inclusion and exclusion criteria has previously been published [14]. None of the subjects had T2D. All study procedures were approved by the institutional review board of the Amsterdam University Medical Center, location AMC, and were in compliance with the declaration of Helsinki. All participants provided written informed consent. Clinical studies were registered in the Dutch trial register (NTR2705, NTR4713, NTR5983, NTR4327).

#### 2.1.2. Replication Cohort

**Figure 1:**
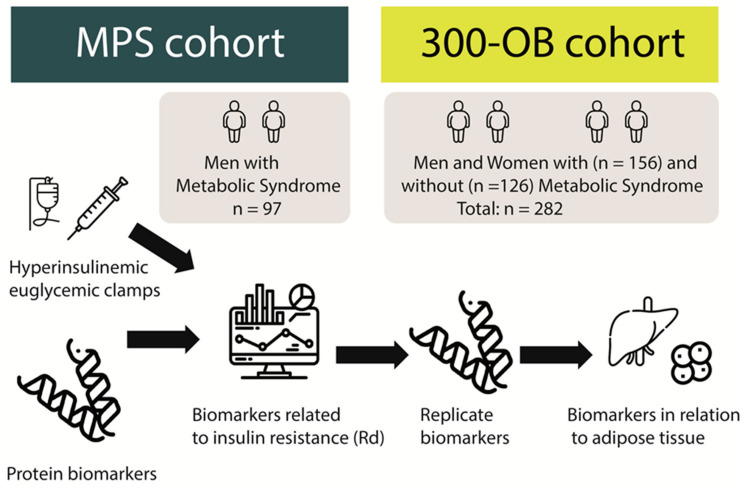
Hyperinsulinemic euglycemic clamps were used in the discovery cohort (Metabolic Profile Study: MPS) in men with metabolic syndrome (n = 97) and protein biomarkers were measured. Using a machine learning and conventional statistics approach, associations between proteins and peripheral insulin resistance (Rd) were assessed. Identified proteins were replicated in the replication cohort (300-OB) cohort with men and women with (n = 156) and without (n = 126) metabolic syndrome. Furthermore, identified proteins were also related to adipose tissue and liver fat in the replication cohort measured by nuclear magnetic resonance imaging.

The validation 300-Obese (300-OB) cohort consisted of individuals recruited in relation to the Human Functional Genomics Project (HFGP, https://hfgp.bbmri.nl/, accessed on 1 September 2019) [15]. Recruited individuals were between 55 and 82 years old, had a BMI > 27 kg/m^2^ and were mostly of Western European descent. Most individuals had previously participated in the Nijmegen Biomedical Study—Non-Invasive Measurements of Atherosclerosis [16]. Information about lifestyle and medication used were gathered via questionnaires. Participants using lipid lowering therapy discontinued their medication four weeks before the measurements to improve comparability between participants. Blood samples were obtained in the morning after an overnight fast. Women were not using any hormonal replacement therapy and all women were postmenopausal. MetSyn was defined as meeting the NCEP-ATP III criteria; see Figure 1. Fasting glucose was used instead of HOMA-IR or plasma insulin as this parameter is more applicable to clinical practice.

### 2.2. Clinical Parameters and Proteins

Measurements of clinical parameters and biochemistry were analyzed in the local hospital laboratory for routine measurements, as previously published [17,18]. Insulin resistance was defined as an insulin-stimulated glucose disappearance rate (Rd) < 37.3 μmol kg^−1^ min^−1^, as previously described [19]. As there is currently no standard definition for insulin resistance defined by clamping, we used this cutoff, which may be arbitrary but has recently been used in similar studies [17,20,21]. No clamp data were available in the replication cohort, and therefore we defined IFG as fasting glucose ≥ 5.6 mmol/L, which is a frequently described measure for IFG [13,22].

Blood samples for proteomics analysis were stored at −80 °C until further processing. Protein concentrations from heparin plasma samples were analyzed by the Olink Proseek Multiplex CVD II and CVD III panel (Olink, Uppsala, Sweden); for more details, see the Supplemental Methods.

### 2.3. Two-Step Hyperinsulinemic Euglycemic Clamp

Insulin sensitivity was measured in all participants of the discovery (MPS) cohort with a two-step hyperinsulinemic clamp with stable isotopes; for detailed methods, see the Supplemental Methods. Glucose fluxes were calculated with the modified Steele equation for (non-) steady measurements [23,24].

### 2.4. Adipose Tissue Analysis

Magnetic resonance (MR) images were analyzed with the HIPPO FAT (v 1.3, v. Positano) in IDL 6.0 [25]. T1-weighted images were used for analysis. Volumes of superficial subcutaneous adipose tissue (sSAT), deep subcutaneous adipose tissue (dSAT), total subcutaneous adipose tissue (SAT) and visceral adipose tissue (VAT) were measured on eight consecutive slices with 5 mm interslice difference at L4-L5; detailed methods have been published previously [26]. 

Hepatic fat content was determined with v3.0 jMRUI using the AMARES algorithm for water (4.7 ppm) and methylene (1.3 ppm) determination [27]. In line with European guidelines, non-alcoholic fatty liver disease (NAFLD) was defined as having a methylene to methylene and water ratio ≥ 5.6% [28]. Detailed methods have been published [26].

### 2.5. Statistical Analysis and Machine Learning Models

Statistical analyses were performed in R (version 3.6.1) and ggplot2 (version 3.5.1) was used for data visualization. Linear regression was used to assess the association between individual proteins and outcome data of interest. To address bias, some models were corrected for potential confounders. Spearman’s test was performed to assess correlations. If data for outcomes were missing, subjects were removed from the analysis. 

For the machine learning models, XGBoost (version 0.90) with classifier and regression models was used, as previously published [20]. In short, fivefold cross-validation and 100 structured iterations were used to ensure robustness and prevent overfitting. Python (v 3.7.4) was used for machine learning models with libraries scikit-learn (v 0.21.2), numpy (v 1.16.4) and pandas (v 0.25.1). Data were split into a test (20%) and training (80%) set during every iteration. For hyperparameters. Optimized models were tested on the training datasets. To ensure the validity of the results, two random variables were added to the predictor data in each iteration to identify irrelevant features.

## 3. Results

### 3.1. Baseline Characteristics

#### 3.1.1. Discovery Cohort

The discovery MPS cohort included 97 men with MetSyn with a mean age of 56 years (SD: 8.0). Within this group, 70 individuals were insulin-resistant based on peripheral insulin resistance (Rd < 37.3 μmol kg^−1^ min^−1^), as recently described to classify insulin-resistant individuals [19]. Weight was higher in the insulin-resistant group but body mass index (BMI) did not differ between the two groups. Other parameters of metabolic health that differed between the groups included insulin and HOMA-IR. Cholesterol levels were not different between the groups (Table 1 and Table S1).

#### 3.1.2. Replication Cohort

The replication cohort (300OB) comprised 302 subjects, of which 282 were eligible for analysis after proteomics quality control. The mean age of this group was 67 years (5.5), and of the group 119 individuals had IFG based on fasting glucose ≥ 5.6 mmol/L. As in the replication cohort, no hyperinsulinemic euglycemic clamps were performed, and IFG was used as a surrogate marker for insulin resistance. BMI and weight were higher in the IFG group (Table 2). In the total group, 159 individuals had MetSyn. In the IFG group, 108 individuals (90.8%) had MetSyn, whereas in the non-IFG group, the number of individuals was 48 (29.4%). In this cohort, 33 individuals had T2D, of which 25 used antihyperglycemic medication, including biguanides (n = 24), sulfonylurea derivatives (n = 13) and insulin (n = 9). In addition to this, 126 individuals used antihypertensive drugs, compared to 156 who did not use this type of drug. Inflammatory parameters such as CRP and leukocyte count were not different between IFG and non-IFG individuals, nor were liver enzymes. In addition to this, some individuals had a history of intermittent claudication (n = 27), percutaneous transluminal coronary angioplasty (n = 10), transient ischemic attack (n = 10), myocardial infarction (n = 5), cerebrovascular accident (n = 4) and heart surgery (n = 1).

### 3.2. Protein Biomarkers Related to Insulin Resistance in Treatment-Naïve Metabolic Syndrome Subjects

#### 3.2.1. Discovery Cohort

**Figure 2:**
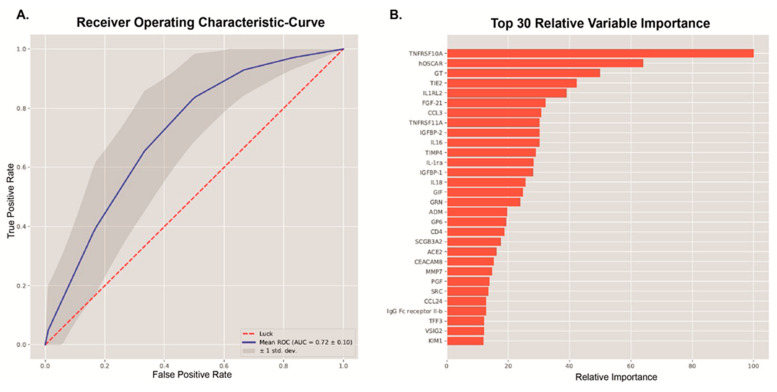
Machine learning approach. (**A**) Receiver operating characteristic curve from the XGBoost classification model to predict insulin resistance (Rd < 37.3 μmol kg^−1^ min^−1^) in the discovery cohort. (**B**) Top 30 most important predictors for the classification of insulin resistance by the machine learning model.

To assess which proteins are related to insulin resistance, we applied a machine learning method to predict which subjects in the discovery cohort had peripheral insulin resistance, based on Rd. We used prediction models to classify which individual was insulin-resistant based on proteins. The classification of peripheral insulin resistance (Rd) in MetSyn was possible based on plasma proteins (AUC = 0.72 ± 0.10, Figure 2). 

To evaluate the individual relation between proteins and Rd, the 30 proteins with the highest feature importance for insulin resistance classification based on Rd were used for linear regression (Figure 2, Tables S2–S4). The linear regression showed that 22 proteins were also individually associated with Rd. After correction for age and BMI, 23 proteins were significantly associated with Rd (Figure S1, Table S5).

#### 3.2.2. Individual Proteins in the Discovery Cohort

**Figure 3:**
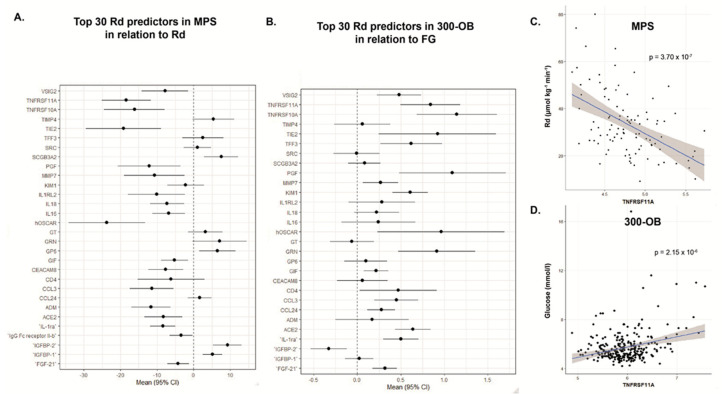
Top 30 classification predictor for insulin resistance based on Rd in relation to clinical outcomes. (**A**) Linear regression between individual proteins predictive for insulin resistance and the rate of disappearance (Rd) in men with metabolic syndrome from the replication cohort (n = 97). A negative association with Rd indicates a relation to insulin resistance. (**B**) Linear regression between individual proteins predictive for insulin resistance and fasting glucose in obese men and women with and without metabolic syndrome (n = 282). A positive association with fasting glucose indicates a relation to impaired fasting glucose. (**C**) Correlation plot of the tumor necrosis factor receptor superfamily 11A (TNFRSF11A) and Rd with the p value of the Spearman correlation test. (**D**) Correlation plot of TNFRSF11A and fasting glucose with the p value of the Spearman correlation test.

Proteins predictive for Rd were related to inflammatory processes such as tumor necrosis factor receptor superfamily member 10A and 11A, C-C motif chemokine 3 (CCL3) and IL-1 family members Interleukin-1 receptor antagonist (IL-1RA) and IL-18, as well as IL-16 and CD40. Some proteins were directly linked to glycemic control, such as insulin-like growth factor binding protein 1 (IGFBP1) and IGFBP2. Other proteins associated with Rd were V-set and immunoglobulin domain-containing protein 2 (VSIG2), osteoclast-associated immunoglobin-like-receptor (OSCAR) metalloproteinase 7 (MMP7), fibroblast growth factor 21 (FGF-21), placenta growth factor (PGF2) and angiotensin converting enzyme 2 (ACE2), Figure 3. Eleven proteins of this subset were also associated with HOMA-IR (Figure S2, Table S6).

### 3.3. Replication of Findings in Obese Men and Women

#### 3.3.1. Replication Cohort

**Figure 4:**
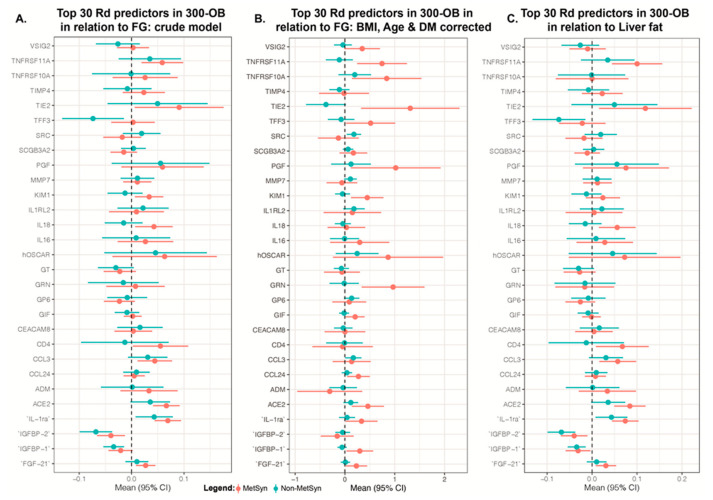
Linear regression between individual proteins and clinical outcomes in the discovery cohort (300-OB) in MetSyn (n = 156) vs. non-MetSyn (n = 126). (**A**) Top 30 protein biomarkers in relation to fasting glucose in the replication cohort (300-OB) crude model. (**B**) Top 30 protein biomarkers in relation to fasting glucose in the replication cohort (300-OB) with correction for potential confounders: BMI, age, diabetes status. (**C**) Top 30 protein biomarkers in relation to liver fat measured by nuclear magnetic resonance imaging in the replication cohort (300-OB) crude model.

As no clamps were performed in the 300OB replication cohort, we used surrogate markers to assess the relation between proteins and glycemic control. As the discovery cohort only consisted of individuals with MetSyn and the replication cohort of individuals with and without MetSyn, we aimed to evaluate whether the proteins predictive for Rd in MPS were similarly related to glucose metabolism in the 300OB cohort, in which individuals were older and had more elevated fasting glucose. The number of individuals with IFG were non-equally distributed between MetSyn and non-MetSyn in the 300OB cohort, and therefore we used glucose as a continuous outcome instead of as a class predictor for impaired IFG. Only 29 proteins from the 30 most predictive proteins for Rd were available after quality control, IgG Fc receptor II-b was not available in this cohort. Using linear regression to evaluate the relation between individual proteins and fasting glucose levels, 18 proteins were significantly related (Figure 3). When only assessing the subgroup with MetSyn (n = 156), fifteen proteins were significantly related to Rd, whereas in the group without MetSyn (n = 126) only two proteins were significantly related to fasting glucose levels (Figure 4, Tables S7–S9). Six proteins were also associated with HOMA-IR (Table S10).

#### 3.3.2. Individual Proteins in the Replication Cohort

In addition to evaluating the overall number of associated proteins, individual proteins provide additional information about IFG in MetSyn. Interestingly, most proteins associated with Rd in the replication cohort were also associated with fasting glucose levels in MetSyn individuals, but not in obese individuals without MetSyn. An example of this is TNFRSF11A, which has a negative association with Rd and a positive one with fasting glucose in the replication cohort. Furthermore, this positive association with fasting glucose was only seen in individuals with MetSyn but not in those without MetSyn. The most apparent group are proteins directly linked to glycemic control, such as IGBP-1. But most frequently associated were proteins related to inflammatory processes such as TNFRSF 10A, TNFRSF 11A and IL-1ra. Furthermore, ACE2, KIM1 and CCL24 were also associated with fasting glucose levels. Correcting for Age, BMI and T2D history yielded similar results (Figure 4B, Tables S11 and S12).

### 3.4. Protein Biomarkers in Relation to Adipose Tissue

To assess if identified protein biomarkers are related to specific adipose tissues, we evaluated the relevant protein to liver fat content, VAT and SAT. The latter was divided into dSAT and sSAT. Liver fat was most strongly associated with fasting glucose (rho 0.36, *p* = 1.79 × 10^−9^), followed by VAT (rho 0.35, *p* = 6.80 × 10^−9^). On the other hand, SAT (rho 0.10, *p* = 0.10^9^), dSAT (rho 0.10, *p* = 0.093) and sSAT (rho 0.02, *p* = 0.696) were not significantly associated with fasting glucose (Figure S3). 

Ten out of thirty proteins were significantly associated with liver fat in men and women with MetSyn, whereas only four proteins were related to liver fat in non-MetSyn obese subjects (Tables S13 and S14). In the MetSyn group, IL-1ra, TNFRSF 11A and ACE2 had the strongest association (*p* < 0.01). In the total group, 13 proteins were related to liver fat and 16 protein biomarkers to VAT (S15–S18). Ten proteins were related to dSAT, of which Il-1ra ADM and TNFRSF11 were the most strongly associated (*p* < 0.01); see Table S19. Eleven proteins were related to SAT, and 13 were associated with sSAT (Tables S20 and S21. IL-1ra and TNFRSF 11A were associated with all assessed adipose tissue types.

## 4. Discussion

In our study, we identified several plasma protein biomarkers related to inflammation and glycemic control which are associated with peripheral insulin resistance, IFG and visceral adipose tissue and liver fat in MetSyn. These proteins were also related to fasting glucose levels in obese men and women with MetSyn, but not in individuals without MetSyn, providing novel insights into metabolic changes in new-onset insulin resistance in MetSyn. To our knowledge, this is the first study that investigated plasma proteins in relation to the gold standard for insulin sensitivity, hyperinsulinemic clamps, in an extensive group of treatment-naïve individuals with MetSyn. As the development of insulin resistance is a delicate process which has been developing over a long period of time and precedes hyperglycemia, sensitive methodologies which have the capacity to detect subtle metabolic changes are necessary to successfully identify early changes which mark the beginning of insulin resistance.

Some proteins which were consistently related to insulin resistance in MetSyn shown by the machine learning approach and conventional statistics are TNF receptors (TNFR). TNF concentrations are also increased in obesity and have been suggested to play a role in the development of insulin resistance [8,29]. In fact, animals with deficient or functionally impaired TNF or TNFR showed improved insulin sensitivity [30,31]. Furthermore, an infusion of TNF induces insulin resistance in healthy humans [32]. However, the mechanism is still not completely understood. In our study, we found that tumor necrosis factor receptor superfamily members 10A (TNFRSF10A) and TNFRSF11A were consistently associated with insulin resistance. This is a receptor for the cytotoxic ligand TNFSF10, also known as TNF-related apoptosis-inducing ligand (TRAIL) [33]. The injection of recombinant human TRAIL in mice reduced inflammatory markers, NAFLD markers [34] and increased insulin-stimulated glucose uptake in mice [35]. In line with this, TRAIL knock-out mice on a high-fat diet have increased insulin resistance, inflammatory cytokines (IL-1 and IL-6) and decreased serum TRAIL levels [36]. This is in line with human studies of newly diagnosed T2D subjects who had decreased levels of serum TRAIL [37]. In accordance with this, in our study, IL-1RA-circulating concentrations were also associated with insulin resistance. However, this difference in TRAIL levels was not present in patients with advanced diabetes resulting in organ damage such as nephropathy or foot ulcers [38]. In addition to this, another study found that newly diagnosed T2D patients have decreased concentrations of serum-soluble TRAIL, whereas this difference was not present after six months of antidiabetic treatment [39]. This suggests that the TRAIL cascade including TNFRSF10A and IL-1RA concentrations represents an adaptive mechanism to counteract the inflammatory changes observed in obesity and metabolic syndrome which are abolished by successful antihyperglycemic therapy. This is also in line with the findings in our study, where we observed that high concentrations of TNFRSF10A were associated with low levels of peripheral insulin sensitivity (Rd) in men with MetSyn without antihyperglycemic medication use. Interestingly, the same pattern was observed in the replication cohort, where MetSyn was also related to high glucose concentrations. In contrast, low concentrations of TNFRSF10A and TNFRSF11A were related to glucose levels in individuals with MetSyn but not in obese individuals without MetSyn. This suggests that metabolic perturbations observed in MetSyn are marked by proinflammatory processes as well as TNF-related receptors. Of note, a similar pattern was observed for interleukins (IL), such as IL-1 receptor 1, IL-16 and IL-18, as well as for chemotactic C-C motif chemokine 3 (CCL3) and CCL24, suggesting that these proteins are part of the initial immune response contributing to perturbed glucose regulation.

In our study, we found that adipose tissue shows different levels of association with fasting glucose depending on the localization. We found that liver fat and VAT are most strongly associated with fasting glucose and that many proteins which can distinguish between insulin-resistant and non-insulin-resistant individuals with MetSyn are related to inflammatory pathways such as TNFRSF10A, TNFRSF11A, IL-1, IL-6 and IL-18. Interestingly, these proinflammatory cytokines are also frequently associated with white adipose tissue inflammation [40]. There is a controversy in the current literature about which adipose tissue compartment contributes most to inflammation and insulin resistance [41]. VAT is heavily studied and has the strongest association with insulin resistance in a meta-analysis of 40 studies [42], but other adipose tissue compartments such as epicardial adipose tissue are also associated with fasting insulin, diastolic blood pressure and other cardiovascular risk factors [43,44]. In addition to this, epicardial adipose tissue is associated with proinflammatory markers such as TNF and IL [45]. In addition to this, perivascular adipose tissue has also been shown to be associated with TNF and CCL2 and different compositions of adipose tissue are observed depending on the location in the body [46]. As there is overlap between proteins and their association with different adipose tissue compartments, it is challenging to speculate on what adipose tissue compartment has the strongest influence on inflammation, in particular because we did not have data on epicardial and perivascular adipose tissue in the present study. However, we found that liver fat and VAT have stronger associations with fasting glucose than dSAT and sSAT. As prolonged low-grade inflammation contributes to the development of insulin resistance [47], it is conceivable that inflammation in liver fat and VAT has the strongest influence on insulin resistance in MetSyn.

The machine learning models showed that proteins directly related to insulin signaling inflammation such as IGFBP-1, IGFBP-2 and TNF receptors, were important features in differentiating between insulin-resistant and non-insulin-resistant MetSyn individuals. But there were also unexpected findings, such as Secretoglobin family 3A member 2 (SCGB3A2) and Angiotensin-converting enzyme 2 (ACE2). SCGB3A2, a protein known to be related to anti-inflammatory immune responses in airway diseases, has also been shown to be associated with the development of prediabetes and diabetes in a prospective French study of 1506 individuals [33]. In line with this, SCGB3A2 was strongly associated with peripheral insulin resistance (Rd) in our study. Another protein highly expressed in the lungs and relevant for energy and inflammation regulation is ACE2. This protein is a newly discovered important part of the renin–angiotensin system, as it degrades angiotensin 2 into angiotensin 1-7, a ligand for the Mas R receptor which effectively has antagonistic effects on angiotensin 2 [48]. Angiotensin I is secreted by adipose tissue and converted into angiotensin 2 by renin and ACE-1 [49]. Interestingly, obese ACE2-deficient mice have increased myocardial insulin resistance, which also increases oxidative stress [50]. Furthermore, angiotensin 1-7 administration in wild-type mice improved insulin sensitivity without effects on body composition or glucose tolerance [51]. In our study, ACE2 was associated with high glucose concentrations consistently throughout the cohort in subjects with MetSyn but not in obese subjects without MetSyn, suggesting that obesity and therefore increased adipose tissue alone is not sufficient for ACE2 induction, but that MetSyn perturbations are necessary, or perhaps that a prolonged exposure to increased adipose tissue might increase circulating ACE2.

The current study provides specific proteins which may contribute to the onset of insulin resistance. However, to infer causality, follow-up studies are necessary. A Mendelian randomization approach focusing on genes related to specific proteins could provide insight into the question if proteins such as TNFRSF10A, IL-1 or IL-16 causally contribute to insulin resistance or if they are a symptom of metabolic changes. In addition to this, in vitro studies should focus on the site of action of novel protein targets. The stimulation of skeletal muscle, white adipose tissue, hepatocytes or β-cells can provide information on what tissue is affected by the identified protein. If successful, findings should be applied in mouse models and eventually human studies to leverage the knowledge of previous studies and treat insulin resistance in humans before metabolic deterioration exacerbates the situation.

### Limitations

This study provides new insights into early metabolic changes in MetSyn, but it also has some limitations. Insulin-resistant MetSyn individuals in the replication cohort showed similar changes in circulating proteins; however, not all biomarkers identified in the discovery cohort were replicated. This could be due to demographic differences between the two groups. The replication cohort was overall older, and glucose levels in the insulin-resistant group were higher, which might be due to beta cell decline instead of IFG processes driven by MetSyn. Furthermore, as the hyperinsulinemic euglycemic clamps to measure insulin sensitivity were only performed in the discovery cohort, it was necessary to use a surrogate marker for insulin sensitivity in the replication cohort. Glucose levels have been widely accepted as a measure for insulin resistance, as prediabetes is defined as having fasting glucose levels ≥ 5.6 mmol/l by the World Health Organization [52], and are more used in clinical practice than HOMA-IR. However, fasting glucose is not the golden standard for measuring insulin sensitivity. It is conceivable that some individuals are insulin-resistant but do not have increased IFG if beta cell reserve capacity is sufficient, which could influence the specificity of identified proteins for insulin resistance in MetSyn. Furthermore, the glucose rate of disappearance (Rd) measured by clamping reflects mainly peripheral insulin resistance, whereas fasting glucose is more influenced by hepatic insulin resistance [20]. A subset used medication in the replication cohort, and as this is only a small subset, we cannot rule out the possibility that this may have influenced the replication of proteins.

## 5. Conclusions and Future Perspective

Early metabolic changes in MetSyn are marked by changes in circulating proteins related to insulin signaling, inflammation and liver fat. Insulin resistance in MetSyn is related to proteins such as IGFBP-2, IL-1, hOSCAR and TNF receptors, which are not seen in obese subjects without MetSyn. These findings provide new insights into the metabolic profile related to insulin resistance and suggest novel protein targets against metabolic changes in MetSyn.

As fasting glucose is an important clinical measure, the inhibition or reversal of insulin resistance would result in improved fasting glucose levels. If the modulation of identified proteins can reverse insulin resistance in vitro in future research, follow-up studies should focus on the modulation of proteins in translational studies to reduce the global burden of insulin resistance and diabetes.

## Figures and Tables

**Table 1:** Baseline characteristics of the discovery (MPS) cohort. Data are presented as mean (SD) or median [IQR].

	Overall	Insulin Sensitive (Based on Rd)	Insulin-Resistant (Based on Rd)	*p*	Test
n	97	27	70		
Age (years)	55.97 (8.01)	55.37 (7.06)	56.20 (8.39)	0.65	
BMI (kg/m^2^)	33.42 [31.00, 35.70]	33.00 [30.55, 34.27]	33.89 [31.26, 36.00]	0.17	nonnorm
Weight (kg)	110.10 [100.00, 122.00]	102.60 [97.30, 111.35]	114.65 [104.20, 123.10]	0.01	nonnorm
Fasting glucose (mmol/L)	5.81 (0.65)	5.68 (0.57)	5.86 (0.68)	0.23	
Insulin (pmol/L)	108.00 [70.00, 138.00]	69.00 [54.50, 93.00]	119.00 [88.50, 143.25]	<0.001	nonnorm
HOMA-IR	3.65 [2.49, 5.10]	2.70 [1.94, 3.25]	4.12 [3.12, 5.20]	<0.001	nonnorm
LDL (mmol/L)	3.50 [2.84, 4.26]	3.10 [2.61, 4.16]	3.50 [2.90, 4.29]	0.20	nonnorm
HDL (mmol/L)	1.12 (0.26)	1.19 (0.33)	1.09 (0.22)	0.09	
Triglycerides (mmol/L)	1.44 [1.17, 1.80]	1.40 [1.13, 1.80]	1.48 [1.18, 1.80]	0.53	nonnorm
Rd (μmol kg^−1^ min^−1^)	31.10 [24.39, 39.33]	49.50 [44.70, 55.48]	27.35 [21.18, 32.77]	<0.001	nonnorm

**Table 2:** Baseline characteristics of the replication (300-OB) cohort. Data are presented as mean (SD) or median [IQR].

	Overall	IFG	Non-IFG	*p*	Test
n	282	119	163		
Age (years)	67.04 (5.34)	67.32 (5.40)	66.83 (5.30)	0.45	
BMI (kg/m^2^)	29.90 [28.30, 31.90]	30.50 [28.65, 32.90]	29.40 [28.00, 31.15]	<0.001	nonnorm
Weight (kg)	88.80 [81.60, 97.80]	90.60 [84.00, 98.95]	86.70 [80.00, 96.75]	0.02	nonnorm
Fasting glucose (mmol/L)	5.72 (1.29)	6.64 (1.53)	5.05 (0.30)	<0.001	
Insulin (pmol/L)	190 [127, 312]	243 [173, 343]	147 [108, 264]	<0.001	nonnorm
HOMA-IR	8.21 [4.91, 13.85]	11.69 [8.39, 17.92]	5.68 [4.04, 10.42]	<0.001	nonnorm
Total cholesterol (mmol/L)	6.30 [5.60, 6.93]	6.26 [5.60, 6.99]	6.30 [5.40, 6.90]	0.68	nonnorm
LDL (mmol/L)	4.13 [3.52, 4.71]	4.12 [3.52, 4.70]	4.14 [3.53, 4.73]	1.00	nonnorm
HDL (mmol/L)	1.29 [1.11, 1.49]	1.21 [1.05, 1.43]	1.35 [1.17, 1.52]	0.001	nonnorm
Triglycerides (mmol/L)	1.62 [1.26, 2.15]	1.88 [1.41, 2.41]	1.47 [1.20, 1.92]	<0.001	nonnorm
Sex = male (%)	122 (43.3)	52 (43.7)	70 (42.9)	1.00	

## Data Availability

Restrictions apply to the availability of some or all data generated or analyzed during this study to preserve patient confidentiality or because they were used under license. The corresponding author will detail the restrictions and any conditions under which access to some data may be provided upon request.

## Supplementary Materials

The following supporting information can be downloaded at: https://www.mdpi.com/article/10.3390/nu16121822/s1, Figure S1: Linear regression between Top 30 predictive proteins and fasting glucose in the discovery (MPS, n = 97) cohort after correction for Age and BMI; Figure S2: Linear regression between Top 30 predictive proteins and HOMA-IR in the discovery (MPS, n = 97) cohort, crude model; Figure S3: Correlation plots between adipose tissue and fasting glucose in all subjects of the replication cohort (300-OB); Table S1: Extended baseline characteristics discover (MPS) cohort; Table S2: Summary statistics linear regression of individual proteins to Rd in the MPS cohort (total group n = 97); Table S3: Summary statistics linear regression of individual proteins to Rd in the MPS cohort (Insulin resistant group n = 70); Table S4: Summary statistics linear regression of individual proteins to Rd in the MPS cohort (Insulin sensitive group n = 27); Table S5: Summary statistics linear regression of individual proteins to Rd corrected for Age and BMI in the MPS cohort (total group n = 97); Table S6: Summary statistics linear regression of individual proteins to HOMA-IR the MPS cohort (total group n = 97); Table S7: Summary statistics linear regression of individual proteins to fasting glucose the 300OB cohort (total group n = 282); Table S8: Summary statistics linear regression of individual proteins to fasting glucose the 300OB cohort (MetSyn group n = 156); Table S9: Summary statistics linear regression of individual proteins to fasting glucose the 300OB cohort (non-Metsyn group n = 126); Table S10: Summary statistics linear regression of individual proteins to HOMA-IR the 300OB cohort (total group n = 282); Table S11: Summary statistics linear regression of individual proteins to fasting glucose corrected for Age, BMI and DM status in the 300OB cohort (metsyn group n = 156); Table S12: Summary statistics linear regression of individual proteins to fasting glucose corrected for Age, BMI and DM status in the 300OB cohort (no-metsyn group n = 126); Table S13: Summary statistics linear regression of individual proteins to liver fat volume in the 300OB cohort (metsyn group n = 156); Table S14: Summary statistics linear regression of individual proteins to liver fat volume in the 300OB cohort (no-metsyn group n = 126); Table S15: Summary statistics linear regression of individual proteins to liver fat volume in the 300OB cohort (total group n = 282); Table S16: Summary statistics linear regression of individual proteins to VAT volume in the 300OB cohort (total group n = 282); Table S17: Summary statistics linear regression of individual proteins to VAT volume in the 300OB cohort (metsyn group n = 156); Table S18: Summary statistics linear regression of individual proteins to VAT volume in the 300OB cohort (no-metsyn group n = 126); Table S19: Summary statistics linear regression of individual proteins to dSAT volume in the 300OB cohort (total group n = 282); Table S20: Summary statistics linear regression of individual proteins to sSAT volume in the 300OB cohort (total group n = 282); Table S21: Summary statistics linear regression of individual proteins to SAT volume in the 300OB cohort (total group n = 282). Reference [53] is cited in the Supplementary Materials.
