# Supplementary material for: Novel Proteome Targets Marking Insulin Resistance in Metabolic Syndrome
Source: Nutrients. 2024 Jun 10;16(12):1822. doi: 10.3390/nu16121822 (PMC11206392; doi:10.3390/nu16121822)
Supplement: Supplementary file 1 [file nutrients-16-01822-s001.zip › Supplemental Methods.docx]

**Supplemental Methods**

**Protein assays**

Blood samples for proteomics analysis were stored at -80°C until further processing. Protein concentrations from heparin plasma samples were analyzed by the Olink Proseek Multiplex CVD II and CVD III panel (Olink, Uppsala, Sweden), which is a multiplex immunoassay for high-throughput detection of cardiovascular related proteins in liquid samples (1). Both panels consisted of 92 proteins which are relevant biomarkers related to cardiovascular diseases or related processes such as immune cell and glucose metabolism (<https://www.olink.com/products-services/target/cardiovascular-iii-panel/> and <https://www.olink.com/products-services/target/cardiovascular-ii-panel/>). Proteins with a standard deviation ≥ 0.2 NPX (AU) or a median deviation ≥ 0.3 NPX (AU) from individuals control samples were excluded.

**Two-step Hyperinsulinemic Euglycemic Clamp**

Insulin sensitivity was measured in all participants of the discovery (MPS) cohort with a two –step hyperinsulinemic clamp with stable isotopes. In short, after overnight fasting blood samples were drawn to assess background isotope enrichment. The stable isotope [6,6-2H2] glucose was then continuously infused until the end of the test (prime: 11 µmol kg^-1^, continuous infusion: 0.11 µmol kg^-1^min^-1^). After a two-hour equilibration period, infusion of insulin (Actarapid, Novo Nordisk Farma B.V., Alphen aan de Rijn, The Netherlands) was started at a rate of 20 mU m^-2^min^-1^. Glucose levels were evaluated every ten minutes by a glucose analyzer (YSI 2300 Stat Plus Glucose Lactate Analyzer, YSI Life Sciences, Yellow Springs, OH, USA). To maintain glucose levels at 5 mmol/l, 20% glucose solution was infused, as previously described (2). Glucose fluxes were calculated with the modified Steele equation for (non-) steady measurements (3, 4).

**References**

1. Assarsson, E.; Lundberg, M.; Holmquist, G.; Bjorkesten, J.; Thorsen, S.B.; Ekman, D.; Lindstedt, P.; Stenvang, J.; Gullberg, M.; Fredriksson, S. Homogenous 96-plex PEA immunoassay exhibiting high sensitivity, specificity, and excellent scalability. *PLoS ONE* **2014**, *9*, e95192.
2. Koopen, A.M.; de Clercq, N.C.; Warmbrunn, M.V.; Herrema, H.; Davids, M.; de Groot, P.F.; Kootte, R.S.; Bouter, K.E.; Nieuwdorp, M.; Groen, A.K.; et al. Plasma Metabolites Related to Peripheral and Hepatic Insulin Sensitivity Are Not Directly Linked to Gut Microbiota Composition. *Nutrients* **2020**, *12*, 2308.
3. Finegood, D.T.; Bergman, R.N.; Vranic, M. Estimation of endogenous glucose production during hyperinsulinemic-euglycemic glucose clamps. Comparison of unlabeled and labeled exogenous glucose infusates. *Diabetes* **1987**, *36*, 914–924.
4. Steele, R. Influences of glucose loading and of injected insulin on hepatic glucose output. *Ann. N. Y. Acad. Sci.* **1959**, *82*, 420–430.
