# Supplementary material for: Novel Proteome Targets Marking Insulin Resistance in Metabolic Syndrome
Source: Nutrients. 2024 Jun 10;16(12):1822. doi: 10.3390/nu16121822 (PMC11206392; doi:10.3390/nu16121822)
Supplement: Supplementary file 1 [file nutrients-16-01822-s001.zip › Supplemental Figures.pdf]

Supplemental Figures

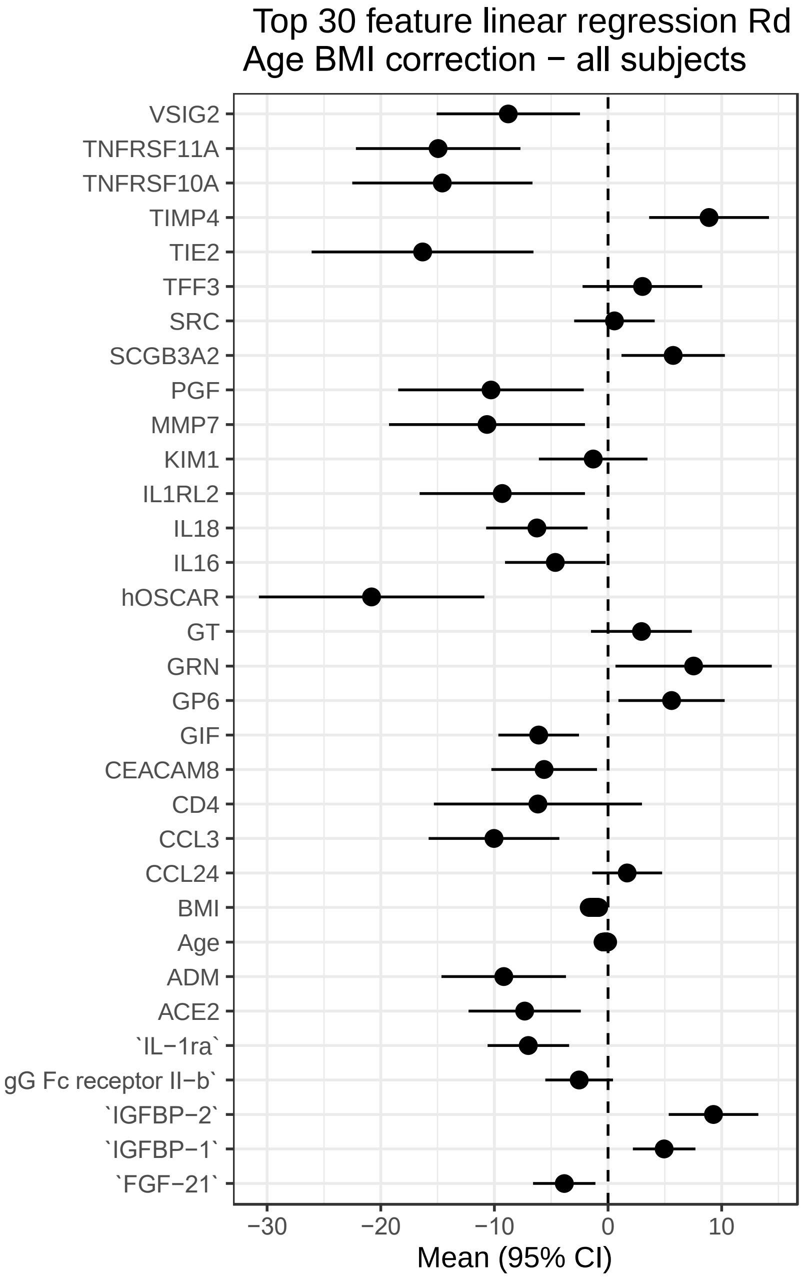

Figure S1. Linear regression between Top 30 predictive proteins and fasting glucose in the discovery (MPS, n = 97) cohort after correction for Age and BMI.

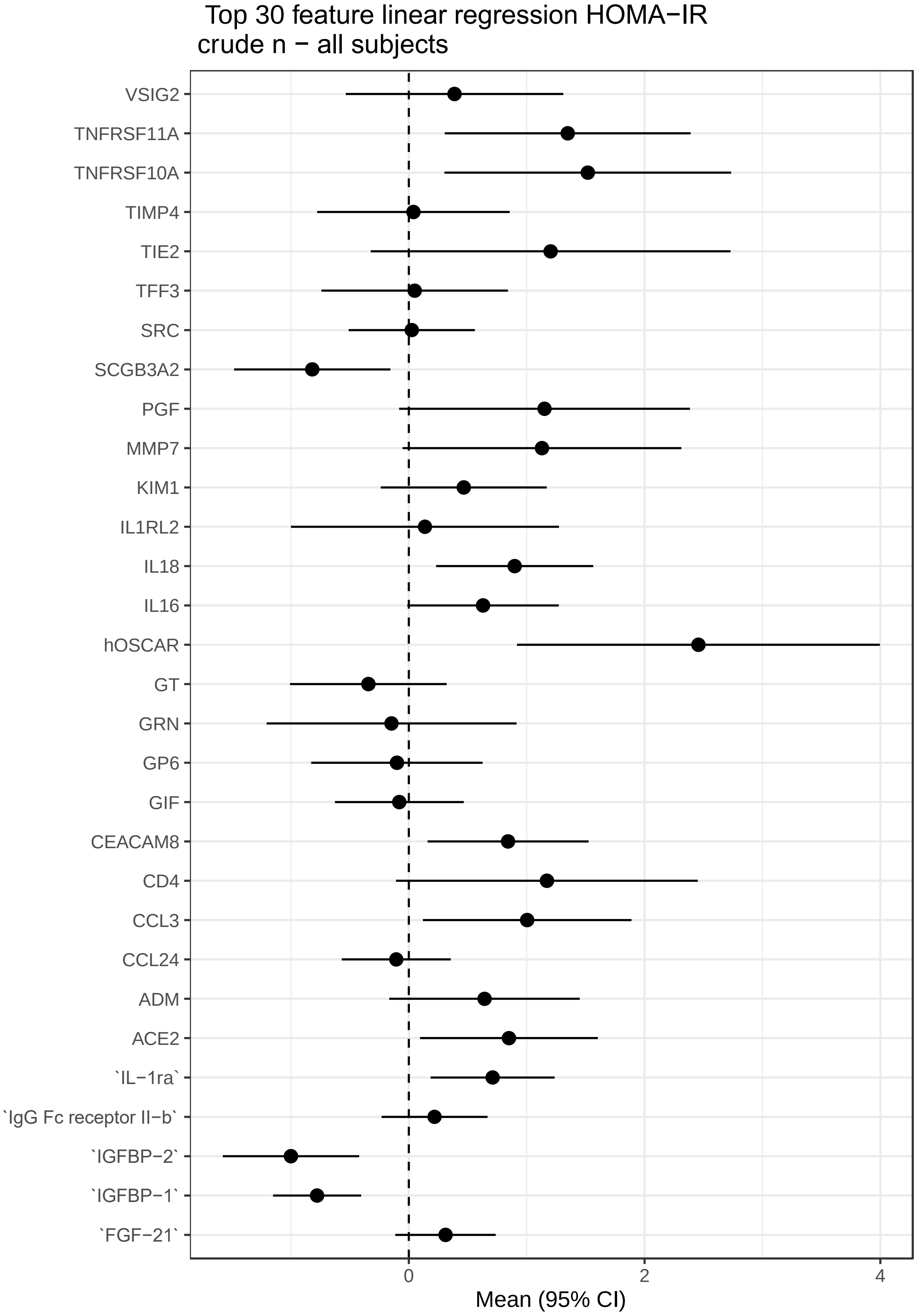

Figure S2. Linear regression between Top 30 predictive proteins and HOMA-IR in the discovery (MPS, n = 97) cohort, crude model.

Supplemental Figures: Novel Proteome Targets Marking Insulin Resistance in Metabolic Syndrome

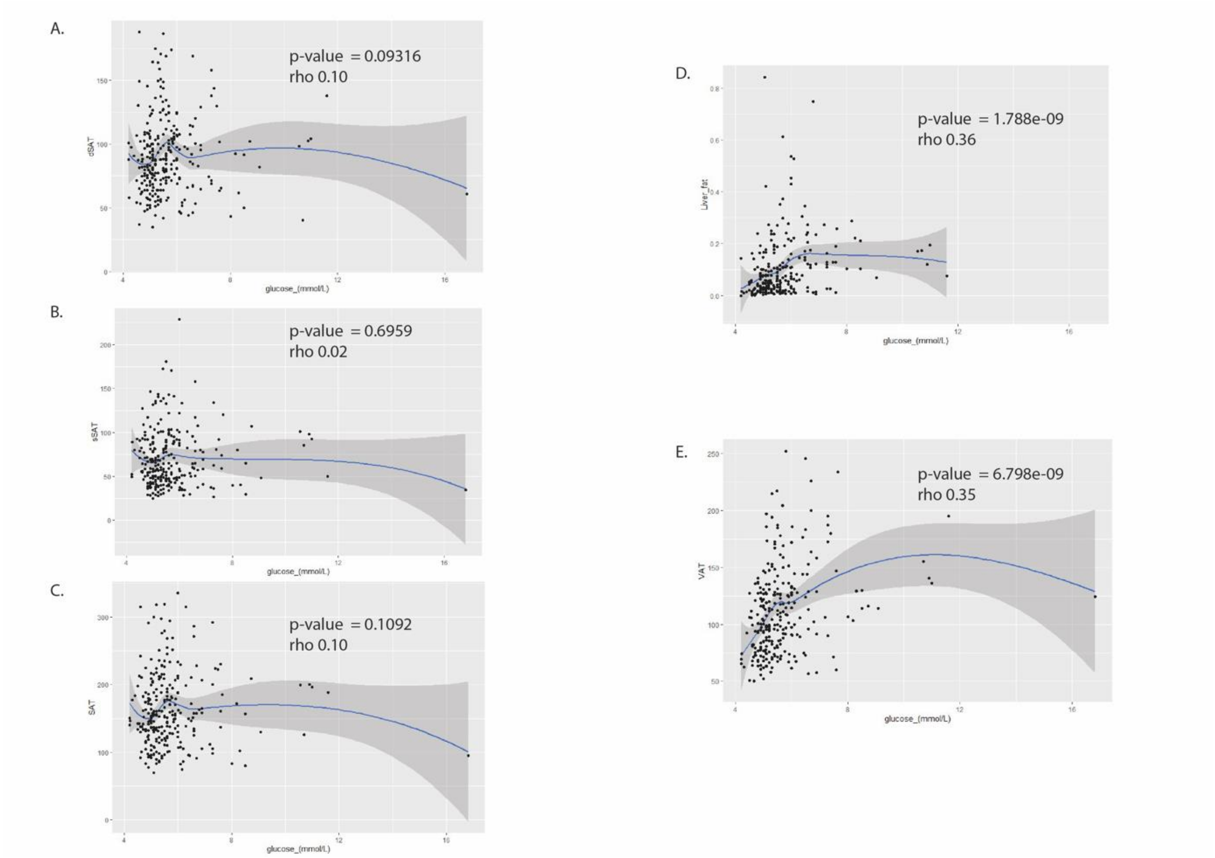

Figure S3. Correlation plots between adipose tissue and fasting glucose in all subjects of the replication cohort (300-OB). A. deep subcutaneous adipose tissue (dSAT) B. superficial subcutaneous adipose tissue (sSAT) C. subcutaneous adipose tissue(SAT) D. Liver fat E. visceral adipose tissue (VAT). Shown p-values and rho are based on Spearman correlation testing.
